# HPE1, an Effector from Zebra Chip Pathogen Interacts with Tomato Proteins and Perturbs Ubiquitinated Protein Accumulation

**DOI:** 10.3390/ijms22169003

**Published:** 2021-08-20

**Authors:** Chia-Cheng Kan, Azucena Mendoza-Herrera, Julien Levy, J. Joe Hull, Jeffery A. Fabrick, Cecilia Tamborindeguy

**Affiliations:** 1 Department of Entomology, Texas A&M University, College Station, TX 77843, USA; cckan@tamu.edu (C.-C.K.); azucena.mendoza@tamu.edu (A.M.-H.); 2 Department of Horticultural Sciences, Texas A&M University, College Station, TX 77843, USA; julienlevy@tamu.edu; 3 USDA-ARS, Arid Land Agricultural Research Center, Maricopa, AZ 85138, USA; joe.hull@usda.gov (J.J.H.); jeff.fabrick@usda.gov (J.A.F.)

**Keywords:** *Ca.* Liberibacter solanacearum, effector, HPE1, ubiquitin-proteasome system, RAD23

## Abstract

The gram-negative bacterial genus *Liberibacter* includes economically important pathogens, such as ‘*Candidatus* Liberibacter asiaticus’ that cause citrus greening disease (or Huanglongbing, HLB) and ‘*Ca.* Liberibacter solanacearum’ (Lso) that cause zebra chip disease in potato. *Liberibacter* pathogens are fastidious bacteria transmitted by psyllids. Pathogen manipulation of the host’ and vector’s immune system for successful colonization is hypothesized to be achieved by Sec translocon-dependent effectors (SDE). In previous work, we identified hypothetical protein effector 1 (HPE1), an SDE from Lso, that acts as a suppressor of the plant’s effector-triggered immunity (ETI)-like response. In this study, using a yeast two-hybrid system, we identify binding interactions between tomato RAD23 proteins and HPE1. We further show that HPE1 interacts with RAD23 in both nuclear and cytoplasmic compartments in planta. Immunoblot assays show that HPE1 is not ubiquitinated in the plant cell, but rather the expression of HPE1 induced the accumulation of other ubiquitinated proteins. A similar accumulation of ubiquitinated proteins is also observed in Lso infected tomato plants. Finally, earlier colonization and symptom development following Lso haplotype B infection are observed in HPE1 overexpressing plants compared to wild-type plants. Overall, our results suggest that HPE1 plays a role in virulence in Lso pathogenesis, possibly by perturbing the ubiquitin-proteasome system via direct interaction with the ubiquitin-like domain of RAD23 proteins.

## 1. Introduction

The bacterial genus *Liberibacter* includes several economically important pathogens, such as ‘*Candidatus* Liberibacter asiaticus’ (Las), ‘*Ca.* L. americanus’, and ‘*Ca.* L. africanus’ that cause citrus greening disease (or Huanglongbing, HLB), as well as ‘*Ca.* L. solanacearum’ (Lso), the causal agent of Zebra chip disease in potato and other diseases. At least seven Lso haplotypes have been reported globally based on different host-vector systems [1]. In the Americas, LsoA and LsoB are vectored by the potato psyllid (also known as tomato psyllid), *Bactericera cockerelli (Šulc)* (Hemiptera: Triozidae) [2,3], and infect solanaceous species.

*Liberibacter* pathogens are phloem-limited, obligate parasitic bacteria. They are transmitted by psyllids, which are phloem-feeding insects, in a persistent propagative manner [4,5]. Manipulations of the metabolism and immune response by these pathogens have been reported in the infected plant hosts, and insect vectors through multi-omic approaches in the past decade. In response to infection, both Las- and Lso-infected host plants undergo shifts in carbohydrate metabolism, and downregulation of photosynthesis- and defense-related genes [6,7,8,9]. On the other hand, in response to *Liberibacter* infections, psyllid vectors show dramatic changes in the expression of genes involved in the citric acid cycle, stress-resistance, and the immune system [10,11]. How these pathogens with simple genomes (~1.2 Mb) manipulate their plant hosts and insect vectors remains unclear.

Accumulating evidence suggests that pathogens use secreted proteins (effectors) to overcome the host immune response and manipulate the host cellular functions to their own benefit. For example, *Pseudomonas syringae* pv *tomato DC3000 (Pst)* effector AvrPtoB was the first E3 ubiquitin ligase-like effector reported to suppress programmed cell death in plants [12]. RipAW and RipAR from *Ralstonia solanacearum* suppress pattern-triggered immunity (PTI) in plants, especially the production of reactive oxygen species, and downregulate the expression of PTI marker genes [13]. Effectors, such as HopBB1 from *Pseudomonas syringae* and HaRxL44 from *Hyaloperonospora arabidopsidis*, use different mechanisms to disturb the ubiquitin/26S proteasome system (UPS), probably by acting as adaptors between the substrates and E3 ligases, resulting in the alteration of phytohormone-related gene transcription and manipulation of plant defenses [14,15].

Unlike the above-mentioned extracellular prokaryotic pathogens, intracellular pathogens, such as Ca. phytoplasma and *Liberibacter*, do not encode syringe-like secretion systems (type III, type IV, and type VI) dedicated for delivering effectors to host cells. Instead, secretion systems utilizing the general secretory (SEC) or twin-arginine translocation (Tat) pathways are assumed to take dominant roles for delivering virulence proteins to their destinations. Phytoplasma effector SAP54 was one of the first reported Sec-dependent effectors of this kind [16]. SAP54 interacts with *Arabidopsis thaliana* flowering related MADS-box transcription factors and the RADIATION SENSITIVE23 (RAD23) family proteins, RAD23c and RAD23d, to mediate the phyllody symptom [17]. SAP54 may play a role as an indirect adaptor regulating the degradation of MADS-box proteins, which represents a new mechanism used by pathogens to perturb proteostasis via the 26S proteasome [18].

To identify potential *Liberibacter* effectors, SEC translocon-dependent secreted proteins were predicted from the Las and Lso genomes by bioinformatic analyses [19,20,21]. The involvement of some of these predicted effectors in plant infection has been determined. For example, SDE1 (CLIBASIA_05315) is more highly expressed when the bacterium is in citrus rather than when present in the psyllid vector. It was determined that SDE1 interacts with members of papain-like cysteine proteases (PLCPs) by yeast two-hybrid (Y2H), and that it inhibits PLCP activity in vitro [22]. CLIBASIA_00460 is also a Sec-dependent protein that is highly expressed in citrus. Heterologous expression of CLIBASIA_00460 in *Nicotiana benthamiana* leads to local and systemic necrosis [23]. Identified by similar approaches, CLIBASIA_00470, CLIBASIA_040250, CLIBASIA_04065c, and CLIBASIA_05150 cause strong cell death symptoms at the systemic leaves in tobacco, and their potential interactors in the host plant were identified by Y2H screening. Understanding the function of Las and Lso Sec translocon-dependent effectors is critical to understand how these intracellular plant pathogens colonize host plants.

In addition to the above-mentioned function of virulence factors, *Liberibacter* effector proteins may also suppress plant defense responses. We previously reported that transient expression of hypothetical protein effector 1 (HPE1) from either LsoA or LsoB suppresses effector-triggered immunity (ETI)-like programmed cell death (HR) induced by Prf^D1416V^ and BAX in *N. benthamiana* [24]. However, how HPE1 suppresses host plant immunity and/or what (if any) factors it may interact with in planta are unknown.

Here, we discovered possible binding partners of HPE1 and attempted to investigate the underlying biochemical mechanisms involved in the suppression of ETI-like immune response by HPE1. We first screened a tomato yeast two-hybrid (Y2H) library and identified RAD23e as a potential HPE1 binding partner. We then confirmed the interaction between HPE1 from Lso haplotypes A and B, and different RAD23 proteins from tomato and the potato psyllid. We further examined the subcellular localization of these proteins, and characterized the potential role of HPE1 in the plant UPS. Lastly, we confirmed a functional role of HPE1 as a virulence factor in planta with transgenic tomato plants over-expressing HPE1. However, the direct functional linkage to the suppression of ETI is still unclear.

## 2. Results

### 2.1. HPE1 Interacts with the Ubiquitin-Like Domain from Tomato RAD23c, RAD23d, and RAD23e

**Figure 1:**
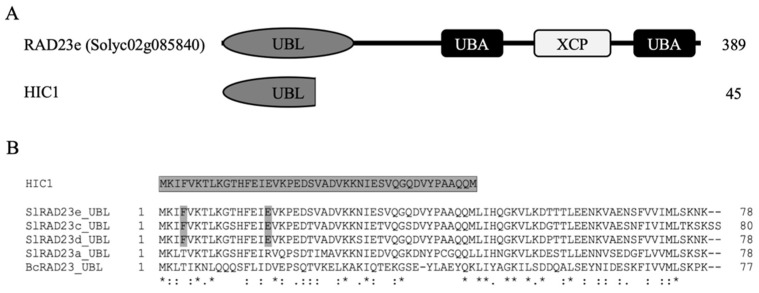
Sequence analysis of the HPE1 interacting clone, HIC1, identified in yeast two-hybrid screening. (**A**) Protein structure of the HIC1 and its full-length encoding protein, RAD23e. (**B**) Amino acid sequence alignment of the ubiquitin-like domain (UBL) of RAD23 proteins from *Solanum lycoperisicum* and *Bactericera cockerelli*. Asterisk, colon and dot denote the conserved and similar amino acid residues.

A prey Y2H library generated by pooling the cDNAs from Lso-uninfected, and LsoB-infected tomato leaves were screened using the HPE1 protein from LsoB as bait. After screening up to 2 × 10^6^ individual yeast clones, we identified 16 clones having three different in-frame insert sequences. Of these, 14 clones had identical coding sequences, which we refer to here as HPE1 Interacting Clone 1 (HIC1). BLASTx analysis against the tomato reference genome (*Solanum lycopersicum* iTAG2.4) revealed that HIC1 corresponds to the first 45 amino acids of Solyc02g085840 (Figure 1A). Solyc02g085840 is RAD23e, a member of the RAD23 protein family that includes three other tomato proteins, RAD23a (Solyc03g117780), RAD23c (Solyc04g007120), and RAD23d (Solyc02g063130) [25]. HIC1 encodes the N-terminal ubiquitin-like domain (UBL) of RAD23e (Figure 1B), a domain that is assumed to interact with the 26S proteasome complex [26]. Because RAD23 serves as the principal shuttle protein for ubiquitinated targets to 26S proteasome, and is conserved in eukaryote species, we hypothesized that a homologous Rad23 gene might exist in psyllids. Therefore, we datamined the *B. cockerelli* transcriptome and identified a RAD23 homolog, which shared 51%, 47%, 51%, and 50% of similarity with SlRAD23a, SlRAD23c, SlRAD23d, and SlRAD23e, respectively (Figure 1B) [10].

**Figure 2:**
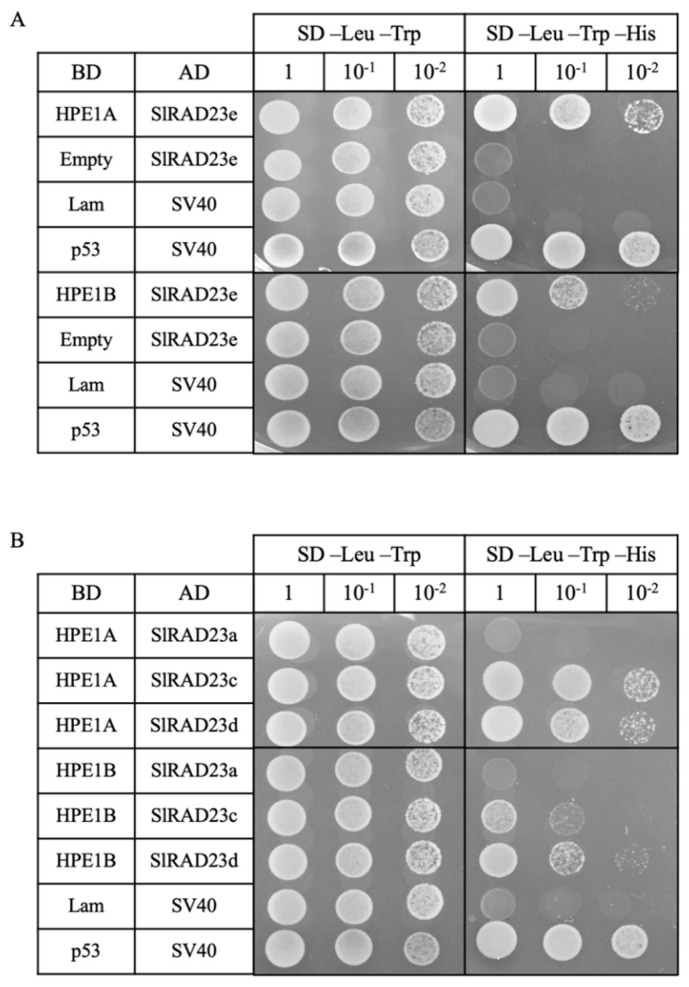
Validation of HPE1 and RAD23 interactions by directed Y2H. (**A**) Y2H test of interaction between SlRAD23e and the HPE1 from LsoA and LsoB. pGBKT7 (BD) constructs were used as bait, and pGADT7 (AD) constructs were used as prey in Y2H. Lam + SV40 indicates negative control for Y2H; p53 + SV40 indicates positive control for Y2H. (**B**) Interaction between HPE1 proteins and the other SlRAD23 proteins.

To evaluate if HPE1 from both Lso haplotypes interacted with the full-length SlRAD23e protein, we performed directed Y2H using either HPE1A or HPE1B as bait, and the full-length SlRAD23e as prey. Our results showed that both HPE1 from LsoA and LsoB interact with SlRAD23e (Figure 2A). Interestingly, the interaction was only observed using less stringent selection conditions (triple nutrient dropout instead of quadruple nutrient dropout as used in Y2H screening), which indicated that the tertiary structure of SlRAD23e protein may affect its relative binding affinity to HPE1.

Because tomato encodes four Rad23 genes, we used Y2H to test if HPE1A and HPE1B interacted with the three remaining RAD23 proteins. Whereas, binding interactions were observed between HPE1 from both haplotypes and SlRAD23c and SlRAD23d, no interaction was observed with SlRAD23a for either haplotype (Figure 2B). Similarly, we also tested if *B. cockerelli* RAD23 interacted with HPE1B and found that neither the full-length BcRAD23, or the BcRAD23 UBL domain alone bound HPE1 (data not shown). These results suggest that HPE1 specifically interacts with the UBL domain of tomato SlRAD23c, SlRAD23d, and SlRAD23e.

### 2.2. HPE1B Colocalizes and Interacts with SlRAD23e in Both Plant Nuclei and Cytosol

**Figure 3:**
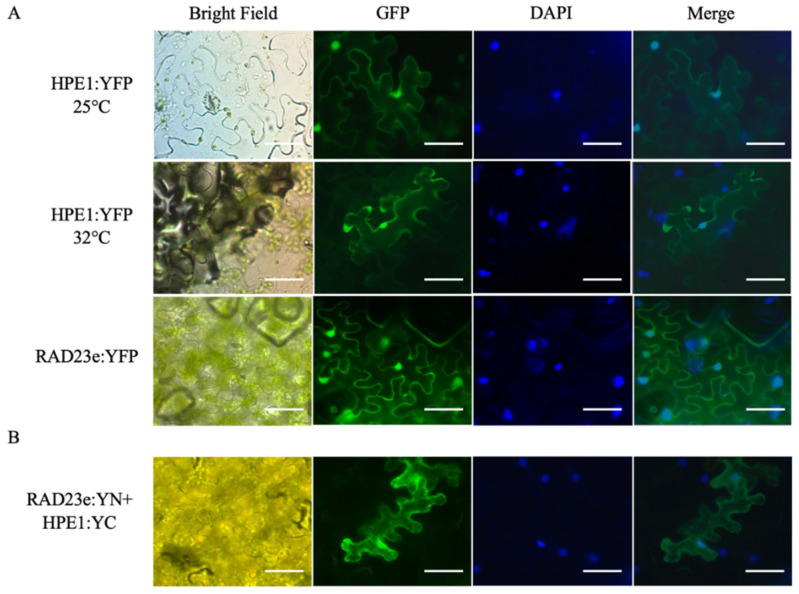
(**A**) HPE1B and RAD23e colocalized at the nuclei and cytosol of *Nicotiana benthamiana* epidermal cells. Upper panel: HPE1B:YFP:HA at 25 °C; Middle panel: HPE1B:YFP:HA at 32 °C; Lower panel: SlRAD23e:YFP:HA at 25 °C. Scale bar = 50 µm (**B**) Bi-molecule fluorescence complementation (BiFC) analysis of HPE1B and RAD23e interaction in *N. benthamiana* epidermal cells infiltrated with a 1:1 ratio of agrobacteria containing HPE1B-YC and RAD23e-YN. Images were taken between 48–60 h postinfiltration.

The subcellular localization of HPE1B and of SlRAD23e were evaluated by transiently expressing each protein fused to a yellow fluorescent protein (YFP) in tobacco leaves. When expressed in tobacco epidermal cells, both HPE1B and SlRAD23e presented a nuclear-cytosolic localization pattern (Figure 3A). In addition, because CLIBASIA_00460, the Las homologous gene of HPE1B, displays a temperature-dependent nuclear accumulation pattern [23]—we, therefore, examined HPE1 subcellular localization at elevated temperature (32 °C). However, unlike CLIBASIA_00460 delocalized from nuclei, HPE1B maintained nuclear localization at higher temperatures (Figure 3A).

Because HPE1B and SlRAD23e colocalized in planta, we investigated the binding of the two proteins by bi-molecule fluorescence complementation (BiFC). Consistent with the colocalization of the individual proteins, a clear YFP signal showing nuclear-cytosolic localization was observed in the BiFC assays (Figure 3B), suggesting a direct interaction between SlRAD23 and HPE1B.

### 2.3. HPE1B and BcRAD23 Colocalize in Cultured Insect Cells

**Figure 4:**
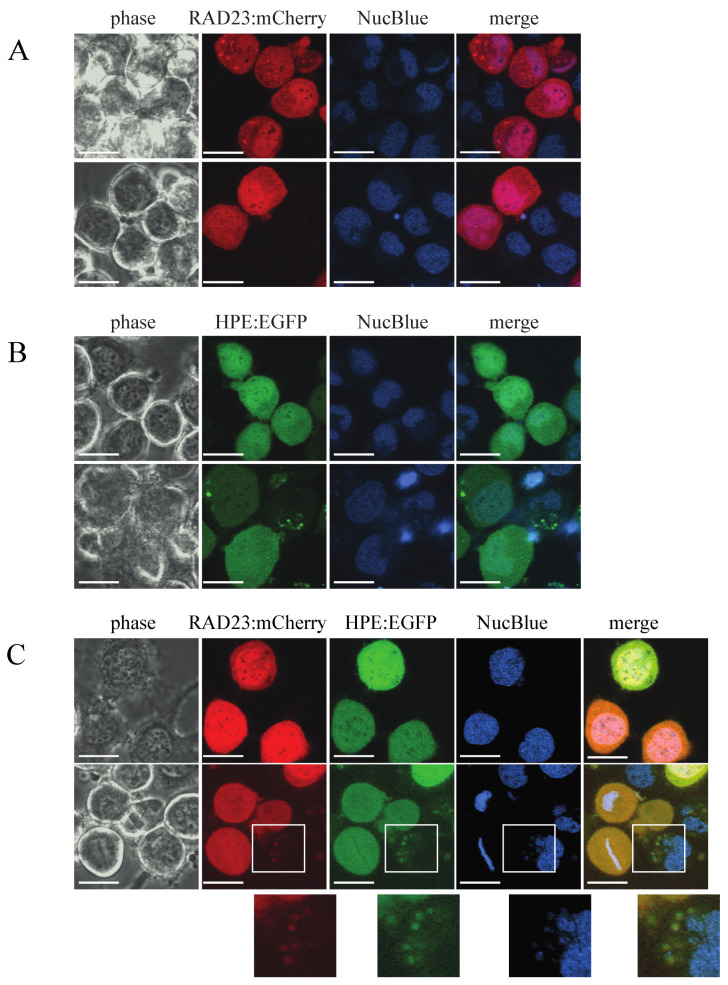
(**A**) BcRAD23e and HPE1B colocalize in the nucleus and cytosol of cultured Tni insect cells. (**A**) Transient expression of a red fluorescent chimera of BcRAD23 (RAD23:mCherry). (**B**) Transient expression of a green fluorescent chimera of HPE1B (HPE:EGFP). (**C**) Colocalization of the RAD23:mCherry and HPE:EGFP fluorescent signals in the cytosol and nucleus. Colocalized signals in discrete punctae were also observed in some cells (lower panel insets). Images were taken 48 h post-transfection with NucBlue used as a counterstain for the nucleus. Scale bar = 20 µm.

To rule out the possibility that BcRAD23 and HPE1B are physically prohibited from binding to each other in a surrogate expressing system, we likewise examined the subcellular localization of BcRAD23 and HPE1B in cultured *Trichoplusia ni* (Tni) insect cells transiently expressing fluorescent chimeras of the two genes. The red fluorescent protein, mCherry, was fused to the BcRAD23 carboxyl-terminus and enhanced GFP (EGFP) was similarly fused to HPE1B. Consistent with the results in *N. benthamiana*, both BcRAD23 (Figure 4A) and HPE1B (Figure 4B) showed clear nuclear-cytosolic localization patterns regardless of being expressed solely or in tandem. In addition to this localization phenotype, puncta in which the two fluorescent signals colocalized were also observed in some cells (Figure 4C). Taken together, our results support that HPE1B and BcRAD23 also colocalize in insect cells.

### 2.4. HPE1B Affects the Accumulation of Ubiquitinated Proteins in Tobacco

**Figure 5:**
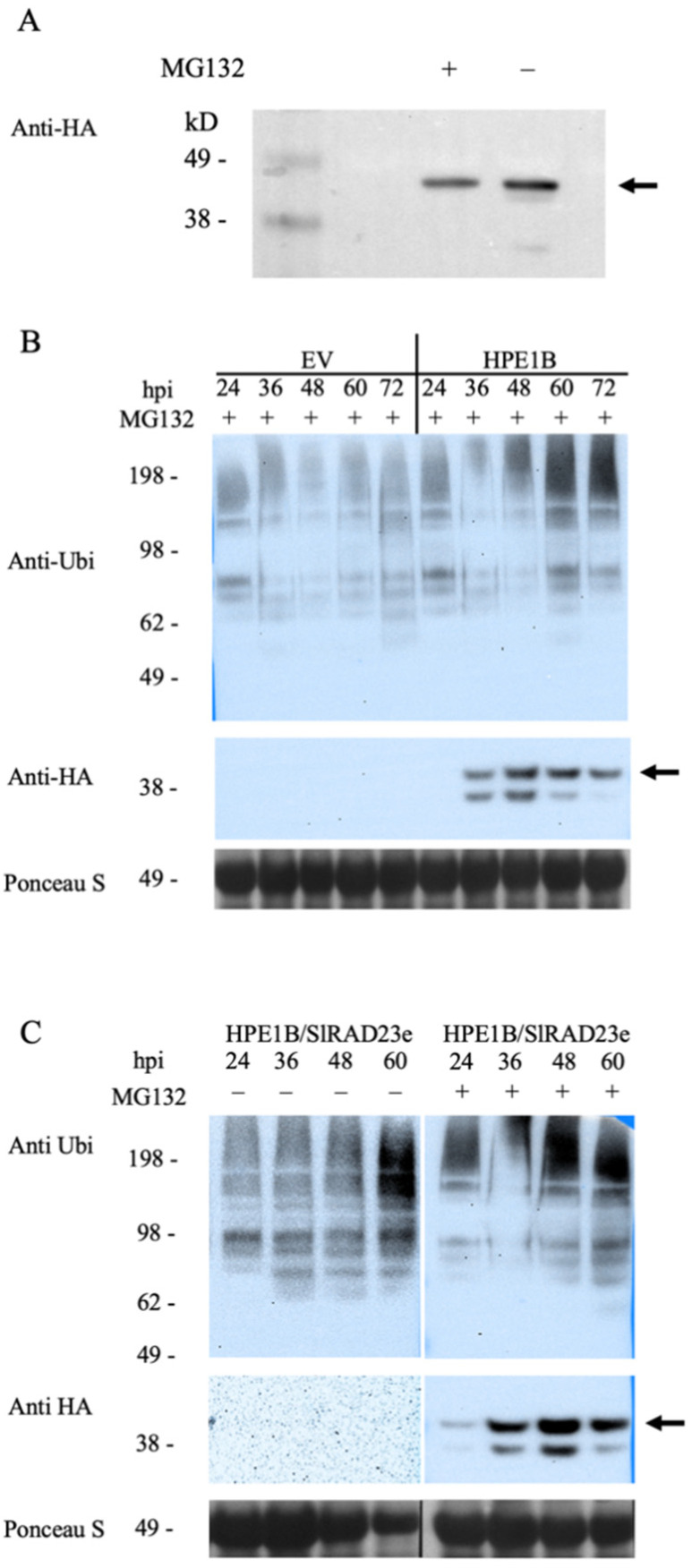
Immunoblot of heterologous expressing of HPE1 in *N. benthamiana*. (**A**) HPE1B is not ubiquitinated in the plant cell, but is degraded in a proteasome-dependent manner. MG132 + and MG132 —indicate treatment of the proteasome inhibitor, MG132. (**B**) Heterologous expression of HPE1B increased the accumulation of ubiquitinated proteins in *N. benthamiana*. HPE1B = HPE1B-YC, EV: empty vector. hpi indicates hours postinfiltration. (**C**) Coexpression of HPE1B and SlRAD23e further decreases the stability of HPE1 proteins in *N. benthamiana*. SlRAD23e = SlRAD23e-YN Arrow indicates the expected size of HPE1B fusion protein.

Since RAD23 proteins have been reported to shuttle ubiquitinated proteins to the proteasome, and thus, facilitate targeted protein degradation, we first suspected that HPE1B is ubiquitinated in plant cells and goes through the typical protein degradation route mediated by the ubiquitin/26S proteasome system. This hypothesis was tested by heterologous expression of the HPE1B protein in planta. When an anti-ubiquitin antibody was used in immunoblot detection, no sign of mono- or polyubiquitinated HPE1 was detected (Figure 5A). However, proteasome-dependent degradation was observed when an anti-HA antibody detected the HPE1B:HA fusion protein.

Next, we examined whether HPE1B expression affected the function of general ubiquitination by binding to the shuttle protein, RAD23, in plant cells. While no ubiquitination of the HPE1B fusion protein was observed as described above, the HPE1B expressing samples consistently showed a higher accumulation of other ubiquitinated proteins when compared with the control samples expressing no fusion protein (Empty vector) (Figure 5B). Moreover, these ubiquitinated proteins continued to accumulate at later time points even after transient expression of HPE1B had peaked at 48 h postinfection (hpi), suggesting that the degradation of HPE1B could be involved in the accumulation of ubiquitinated proteins.

Interestingly, when coexpressed with RAD23e, HPE1B appeared to be less stable as the immunoblotting could not detect the protein without the proteasome inhibitor, MG132 (Figure 5C). Overall, detection of ubiquitinated proteins by immunoblot revealed the possible inhibition of the normal shuttling function of RAD23 proteins and a decreased stability of HPE1B, resulting in the accumulation of ubiquitinated proteins in plants.

### 2.5. Lso Infection Results in the Accumulation of Ubiquitinated Proteins

**Figure 6:**
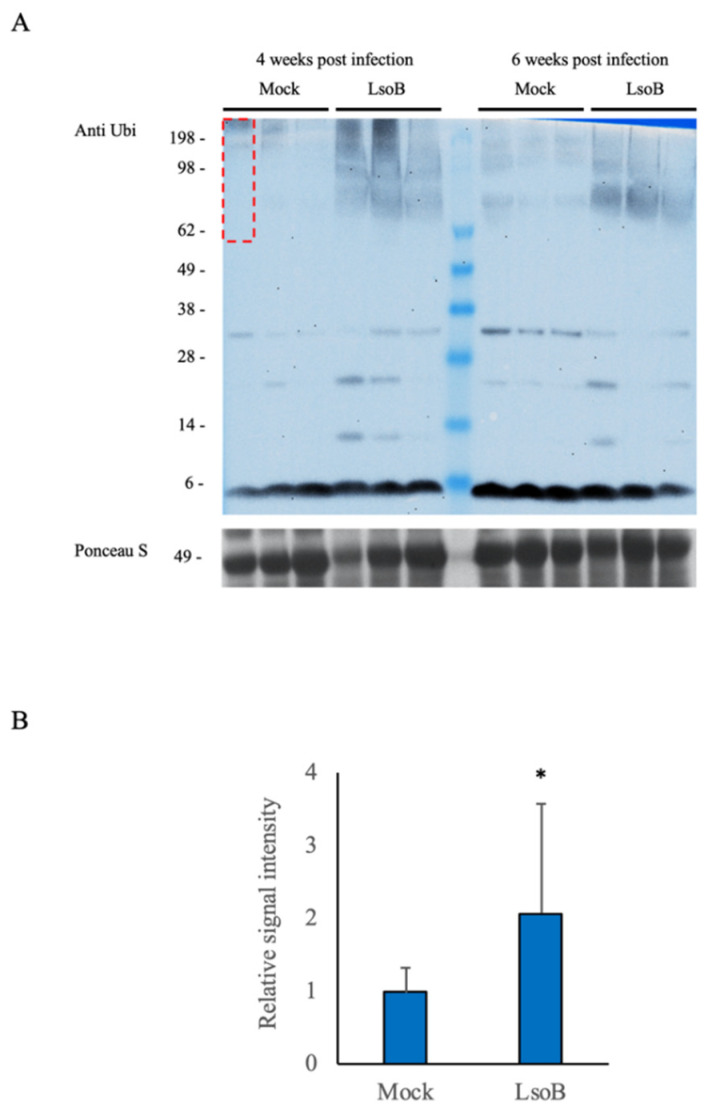
Immunoblot of tomato leaves at 4 weeks and 6 weeks post-LsoB infection. (**A**) Plants were infested with either Lso-free (Mock) or LsoB-infected (LsoB) adult psyllids. The dotted box indicates the range (60–200 kDa) used to quantify the ubiquitin signal intensity in (**B**). Error bars indicate the standard deviation (SD). Asterisk indicates significant difference from Mock group by Student’s *t*-test (*p* < 0.05). The experiment was replicated three times, each with three plants in each group. The Western blot was repeated at least twice per sample, each showing similar results.

We further investigated whether Lso infection leads to a disturbance in UPS in tomatoes. A difference in the abundance of ubiquitinated proteins was observed in the upper leaves of tomato plants at 4 and 6 weeks postinfection (Figure 6A). When the ubiquitin signal was quantified for the protein size ranging between 60 kDa to 200 kDa, a statistically significant difference was observed between the LsoB-infected and mock–inoculated groups (Figure 6B).

### 2.6. Overexpressing HPE1B Facilitates LsoB Colonization and the Development of Symptoms

To further investigate the function of HPE1 on its host plant, transgenic tomatoes expressing HPE1B were generated. We specifically included both the full-length and the mature protein of HPE1B for accessing the complete effects that HPE1B may have on tomato plants. For both types of HPE1B transgenic plants, the expression of HPE1B was confirmed at both the RNA and protein levels (data not shown). No apparent defects in plant growth and development were observed in T_0_ plants. These T_0_ plants were then inoculated with LsoB, and the progression of Lso colonization was determined based on the PCR detection method [27], and the development of symptoms.

**Figure 7:**
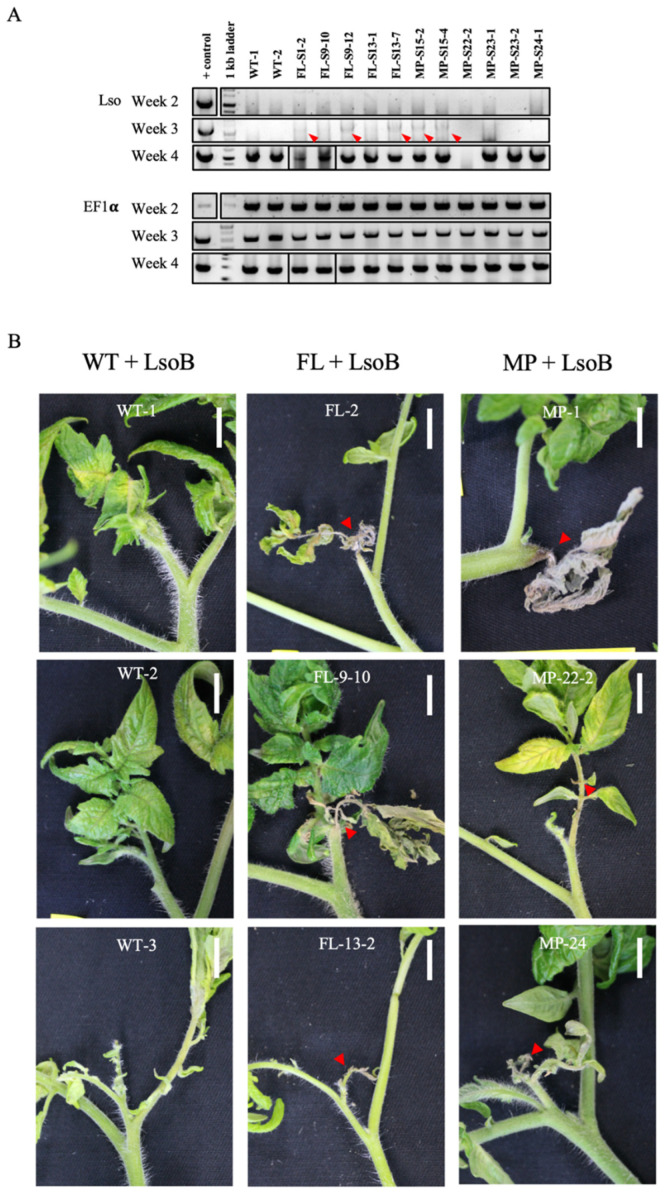
Lso colonization in wild-type and HPE1 transgenic tomato plants. (**A**) PCR detection of Lso at 2-, 3-, and 4-week postinfection. WT, wild-type; FL, full-length HPE1B; MP, mature protein HPE1B. Arrowhead indicates the expected band of the Lso PCR product. (**B**) Symptom development was examined at 7 weeks postinfection. Arrowhead indicates the necrosis symptom of the shoot tip observed. Scale bar = 1 cm.

Interestingly, LsoB was detected in more T_0_ plants (58.8%, 34 plants) than in wild-type plants (46.2%, 13 plants) at three weeks postinfection (Figure 7A). Furthermore, the typical progression of symptoms for LsoB infected plants, which begins at seven weeks postinfection, manifests with stunting and leaf discoloration, then transitions to leaf necrosis, and finally plant death. In three independent infection experiments, the onset of stunting and necrosis, due to infection at the shoot tip in the transgenic plants occurred earlier than the typical seven weeks postinfection in the wild-type plants (Figure 7B). Together, our results indicated that HPE1 is a positive virulent factor that can facilitate LsoB colonization in tomatoes, as well as accelerate the onset of perceived symptoms, possibly through the perturbance of SlRAD23 functions.

## 3. Discussion

Despite the substantial global economic impact of Liberibacter pathogens, our understanding of the molecular mechanisms involved in host infection and disease progression remains superficial. Although novel methods for screening antimicrobial compounds have been developed [28,29], increased understanding of the virulent mechanism can reveal precise targets in the pathosystems for creating new disease control strategies. More than 200 potential effectors have been reported from Liberibacter pathogens, but only about a dozen have been functionally characterized. In this study, we extend our findings that the Sec translocon-dependent effector, HPE1, is involved in evading and/or suppressing the ETI-like immune response in tobacco. We investigated the effector by identifying the potential molecular targets of HPE1 in tomatoes by using the Y2H approach, and then examined the possible function of HPE1 on proteasome degradation. We also showed that it is likely that impaired RAD23 function by HPE1 over-expression resulted in perturbation of UPS. Lastly, we found that LsoB colonization was facilitated on HPE1B over-expressing transgenic tomatoes, which provided evidence connecting HPE1 to the virulence of Lso.

We identified a prey clone, encoding the partial UBL domain of RAD23, interacting with HPE1 by Y2H screening. One of the pitfalls of Y2H is identifying false positive interactions, due to forced heterologous expression in the yeast cells. To strengthen the confidence of observed interactions between HPE1 and SlRAD23 proteins, and gain information on the subcellular localization of these proteins, we performed agroinfiltrations to transiently express these target proteins. Results from the agroinfiltration and BiFC confirmed the colocalization and interaction of these two proteins in the cytosol and the nucleus. Transient expression by agroinfiltration has been widely applied in plant systems and was used to discover temporal-spatial changes of proteins in vivo. For example, CLIBASIA_00460, the homologous gene of HPE1 in Las, was found to exhibit temperature-dependent nuclear translocation [23]. CLIBASIA_00460 is a potential virulence factor that positively correlated with Las pathogenicity [30]. Similar to HPE1, consistent expression during pathogen infection was detected for CLIBASIA_00460 [31]. However, the two SDEs do not share the temperature-dependent-nuclear translocation pattern (Figure 2A). This may be explained by the low sequence similarity of HPE1 and CLIBASIA_00460 (60% amino acid sequence similarity). It is possible that these two genes have evolved host-specific functions, and it is worth investigating the potential interactors of CLIBASIA_00460 in citrus.

RAD23 is a broadly conserved protein family; it consists of three to eight members in eudicot plant species: Four members in Arabidopsis (AtRAD23a–AtRAD23d) [26], and four members in tomato (SlRAD23a–SlRAD23e) [25]. The main cellular function of RAD23 is to shuttle ubiquitin-conjugated proteins to the 26S proteasome. This function is achieved by the four conserved domains in RAD23 proteins: One UBL domain, one XCP domain, and two UBA domains. It is believed that UBA acts as a substrate-binding domain to recruit polyubiquitinated substrate proteins, while UBL is the interacting domain with RPN1 or RPN10 of the 26S proteasome complex, and thus, helps release the polyubiquitinated substrates for degradation. As a result, RAD23 facilitates the protein turnover of selected substrates. Tomato Stress-Associated Protein 4 (SlSAP4) is a known substrate of SlRAD23, that binds to the first UBA domain, possibly resulting in the degradation of substrate proteins involved in reactive oxygen species (ROS) generation [25]. Direct interaction of Arabidopsis RAD23 proteins with the phytoplasma effecter SAP54 was reported and resulted in the degradation of substrate MADS box transcription factors that are related to the phyllody symptom [17]. Unlike SlSAP4, which binds the UBA domain, our results showed that HPE1 interacts with the UBL domain of SlRAD23e. Furthermore, HPE1 selectively interacts with RAD23c, RAD23d, and RAD23e, suggesting that HPE1 does not interact with RAD23 as a ubiquitin receptor mimic, since the hydrophobic patch (Leu-8, Ile-44, Val-70) critical for RPN10 binding is conserved in all SlRAD23 proteins. In yeast, RAD23 interaction with the proteasome is regulated by the phosphorylation status of the UBL domain, and the substrate turnover was inhibited when the RAD23-proteasome interaction was perturbed [32]. This may explain the accumulation of ubiquitinated proteins we observed in the transient expression of HPE1 in tobacco.

Another possible role of HPE1 is in disruption of the mechanism that RAD23 uses to evade proteasome-mediated degradation. RAD23 proteins can escape degradation because they lack an appropriate proteasome initiation region [33]. An addition of 44–102 amino acids after the UBL domain led to the initiation of degradation. The HPE1 mature protein is 99 amino acids in length. By binding to RAD23, HPE1 may serve as the initiation region for degradation of the HPE1-RAD23 complex. A similar mechanism may also explain the initiation of AtMIN2 (RAD23 homolog) degradation when coexpressed with *P. syringae* pv. *tomato* DC3000 effectors, HopM1 and AvrE [34]. Further study on the stability of RAD23 in the presence of HPE1 will be needed to test this hypothesis.

Emerging evidence shows that RAD23 plays important role in the colonization of hosts by pathogens and modification of plant immunity to multiple pathogens. Phytoplasma is the most extensively studied case where the pathogen manipulates AtRAD23 to reshape flower development to the pathogen’s benefits [17]. Silencing of RAD23 in barley was found to decreased the secondary hyphal growth of the powdery mildew pathogen, suggesting a positive regulating role of the pathogenesis [35]. AtRAD23 was suggested to be involved in the regulation of Arabidopsis susceptibility to the non-pathogen *P. syringae* pv. *phaseolicol*, possibly by promoting target degradation in collaboration with AtSAP9 [36]. In tomatoes, SlSAP4 interacts with RAD23 proteins and controls the generation of ROS that leads to localized plant cell death, which is favorable to the colonization by the necrotrophic pathogen *Botrytis cinerea*, but not the biotrophic pathogen *P. syringae* pv. *tomato* DC3000 [25]. In our study, we showed that the turnover of ubiquitinated proteins in plants expressing HPE1 was perturbed, possibly due to the direct interaction of HPE1 and RAD23. We also showed that HPE1 overexpressing transgenic plants promotes the colonization of at least one haplotype of Lso. It would be intriguing to study whether the function of the proteasome is attenuated, due to HPE1 and RAD23 interaction.

As described by Üstün et al. [37], the 26S proteasome is largely involved in plant immunity, particularly in defense response signaling. Many plant immune signaling factors, such as the PAMP receptor FLS2, the regulator of salicylic acid-dependent defense NPR1, or the transcription factor WRKY45, are degraded through specific E3 ubiquitin ligases and the 26S proteasome pathway. Besides, several proteasome subunits have been identified to contribute directly to defense responses, such as ROS production and HR development. The β1 subunit of the 20S core proteasome (CP) was shown to be a negative regulator of early plant responses to the fungal elicitor, cryptogein [38]. PBA1, the main catalytic subunit of the CP, was found to act as a caspase-like enzyme during the induction of programmed cell death [39]. RPN1a, a component of the 19S regulatory particle, was found to be required for resistance against biotrophic fungi. A critical intracellular innate immune receptor protein RESISTANCE TO PSEUDOMONAS MACULICOLA1 (RPM1) for *P. syringae* effector-triggered HR is well known to be dependent on the proper 26S proteasome-mediated degradation to be functional. Considering the broad involvement of UPS in the plant immune system, it is not surprising that plant pathogens exploit effectors that target the UPS to their benefit [37,40,41].

Although no effector-triggered immunity has been identified for Liberibacter pathogens to date [42], our previous identification of HPE1 was one of the first reported suppressors of the ETI-like immune response from phloem inhabiting pathogens. In this study, we attempted to investigate the mechanism behind the immune suppressor role of HPE1. However, from our current results, it is still not clear how the expression of HPE1 in plants inhibits Prf^D1416^ triggered HR cell death. It is possible that polyubiquitinated HR signaling components are not shuttled properly to the proteasome, due to the HPE1-RAD23 interaction. It will be important to further identify the possible substrate proteins of the HPE1-RAD23 complex involved in the plant defense pathway. We proposed that approaches, such as yeast-three hybrid screening [43] and ubiquitylome screening [44], can extend our understanding of the fine manipulation of host proteostasis during Lso infection. Last, but not least, we showed that expression of HPE1B in transgenic tomato plants enables the progression of Lso infection, and further studies using different Lso haplotypes may enhance our understanding of the underlying mechanisms of Lso infection.

## 4. Materials and Methods

### 4.1. Plant and Psyllid Populations for Lso Infection

Tomato (*Solanum lycopersicum* L. ‘Moneymaker’; Thompson and Morgan Inc., Jackson, NJ, USA) plants and tobacco (*Nicotiana benthamiana*) were grown from seed in pots with Sun Gro Sunshine LP5 mix (Bellevue, WA, USA) and fertilized twice a week with Miracle-Gro Water-Soluble Tomato Plant Food at the label rate (18-18-21 NPK; Scotts Miracle-Gro Company, Marysville, OH, USA). One-week-old seedlings were transplanted to individual pots and let grow in the same conditions.

Lab-reared ‘*Ca*. Liberibacter solanacearum’-free and LsoB-infected psyllid populations were maintained separately on tomato plants in insect-proof cages (24 by 13.5 by 13.5 cm; BioQuip, Compton, CA, USA) at room temperature (24 °C) and under a photoperiod of 16 h light- 8 h dark, as described previously [45]. These psyllid populations are henceforth referred to as Lso-free, and LsoB populations, respectively.

For Lso infection, five-week-old tomato seedlings were used, and four adult psyllids from the desired population were mesh-bagged with a lower-tier leaf for seven days before being removed. Each infection experiment had at least three plants, and the experiments were repeated at least three times.

### 4.2. RNA Extraction

One hundred mg of leaf samples from the top-tier leaves were collected and flash-frozen in liquid nitrogen for storage at −80 °C before extraction of RNA. Trizol reagent (Invitrogen, Carlsbad, CA, USA) was used for the RNA extraction following the user’s manual. TURBO DNA-free (Life Technologies, Carlsbad, CA, USA) was used for removing DNA. For detection of gene expression, a Verso cDNA synthesis kit (Life Technologies, Carlsbad, CA, USA) was used to generate first-strand cDNA.

### 4.3. Y2H Library Preparation

A cDNA library was constructed using the Make Your Own “Mate and Plate” Library System (Takara Bio, San Jose, CA, USA) following the user’s manual. Briefly, RNA from Lso-free-, and LsoB-infected tomato leaves were obtained and pooled to increase the transcript representation. Double strand cDNA was generated from 1 μg of pooled tomato RNA by ‘SMART’ MMLV Reverse Transcriptase with SMART III Oligo and CDSIII/6 primer. The cDNA pool was amplified by long-distance PCR (LD-PCR) using Advantage 2 PCR Premix (Takara Bio, San Jose, CA, USA) with the following conditions: Initial denaturation step at 95 °C for 30 s; followed by 20 cycles of 95 °C for 30 s, 68 °C for 6 min with 5 s increment of extension time per cycle; then a final extension step at 68 °C for 5 min. Size selection of the cDNA was made by Chroma Spin TE-400 column. The resulting cDNA inserts were cloned into prey vector by an in vivo recombination approach—4 μg of tomato cDNA was cotransformed with 3 μg of linearized pGADT7-Rec vector into yeast (*Saccharomyces cerevisiae*) strain Y187 following the user’s manual of Yeastmaker Yeast Transformation System 2 (Takara Bio, San Jose, CA, USA) and plated on leucine dropout minimal medium (SD−Leu). After four days of incubation at 30 °C, yeast cells were harvested using a freezing medium (YPDA+ 15% glycerol). The quality of the cDNA library was evaluated based on the colony-forming unit (cfu) on the selection medium and by the final titer of the pooled library.

### 4.4. Yeast Two Hybrid Screening and Directed Y2H

The sequence encoding the mature protein (without signal peptide) of HPE1B (Accession number: WP_013462289) was cloned into the *Eco*RI and *Bam*HI sites of a pGBKT7 vector. The construct was sequenced and confirmed prior to transformation into the Matchmaker Y2H Gold yeast strain. The bait strain was cultured in SD−Trp medium and brought to a final concentration of > 10^8^ cells/mL. Mating was performed by combining 5 mL of bait cells with 1 mL of prey library (>2 × 10^7^ cells/mL for each screen) supplemented with 2 X YPDA media at 30 °C for 24 h at 35 rpm. Mated cells were collected and resuspended in sterilized water and plated on a selective quadruple dropout medium (SD −Leu/−Trp/−His/−Ade, QDO). After incubation at 30 °C for 7 days, yeast colonies that grew well were subjected to colony PCR following the method described in the Yeast Protocol handbook (Clontech, San Jose, CA, USA), and the PCR products were sequenced. Positive colonies were transferred to a new selection medium to confirm growth and interaction intensity. Plasmids of these positive clones were rescued according to the Matchmaker Library Construction and Screening Kits (Takara Bio, San Jose, CA, USA) protocol and subjected to sequencing. The insert sequences for these positive clones were used to BLAST against the tomato genome (iTAG2.4) using the Phytozome v12 database (https://phytozome.jgi.doe.gov/pz/portal.html, accessed date: 25 July 2020).

For directed Y2H, full-length SlRad23 and *Bactericera cockerelli* Rad23 coding sequences were cloned into the GADT7 vectors as prey, and the coding sequence of the mature HPE1A protein was cloned into pGBKT7 as bait. The desired pair of prey and bait constructs were cotransformed into Y2H Gold yeast and plated on SD−Leu−Trp (DDO) medium. Yeast clones with paired constructs were cultured, and 10 μL droplets of the culture dilutions were transferred onto DDO, SD−Leu−Trp−His (TDO), and QDO with a supplement of 40 μg/mL X-alpha-Gal.

### 4.5. Agroinfiltration and Bi-Molecular Fluorescence Complementation

The HPE1B coding region for mature protein was amplified from DNA extracted from psyllids harboring LsoB. The full-length CDS of the SlRad23e gene was amplified from tomato cDNA. PCR was performed using Phusion High-Fidelity DNA Polymerase and gene-specific primers described in Supplementary Table S1. The purified amplicons were TOPO cloned into the pENTR vector according to the manufacturer’s directions (Thermo Fisher Scientific, Waltham, MA, USA) and verified by sequencing. The purified pENTR vector containing HPE1B and Rad23 genes was used to subclone into a YFP containing binary expression vector, pEarlryGate101-YFP, with the Gateway LR Clonase II enzyme (Thermo Fisher Scientific, Waltham, MA, USA). The resulting constructs pHPE1:YFP and pRAD23e:YFP were transformed into *Agrobacterium tumefaciens* strain LBA4404 by electroporation.

For BiFC assays, previously cloned pENTR constructs were used to subclone the RAD23e coding sequence into the pEarleyGate201-YN vector, and HPE1B (without signal peptide) was cloned into pEarleyGate202-YC using LR Clonase II. The full-length YFP function is complemented when targeted proteins physically interact with each other, as described by Lu et al. [46]

Transformed *A. tumefaciens* were grown at 28 °C for 16–24 h, in the LB medium containing 10 mM morpholinnehanesulfonic acid (MES) pH 5.6 and 20 μM acetosyringone (AS). The cultures were pelleted and resuspended in freshly prepared infiltration buffer (10 mM MgCl_2,_ 10 mM MES, and 200 μM AS) to a final OD_600_ = 0.8. Cells were incubated in the dark for at least 4 h at room temperature. *Agrobacterium* carrying pEarleyGate101 or BiFC constructs (coinfiltration at *v*/*v* ratio = 1:1) infiltrated into the intercellular spaces the leaves of 4-week-old *N. benthamiana* plants with a needleless syringe. For temperature-dependent localization assay, *N. benthamina* plants were transferred to a controlled growth chamber set at 32 °C for three days before agroinfiltration. After infiltration, the plants were maintained at 32 °C until microscopically examined using a fluorescent microscope (Axio Imager A1 microscope, Carl Zeiss Microscopy, White Plains, NY, USA) with a FITC (488 nm, green) filter for YFP signal.

For immunoblot assays, leaf samples were collected at 24, 36, 48, 60, 72 h after agroinfiltration using a cork borer (diameter = 1.5 cm). Proteasome inhibitor, MG132 (final concentration = 50 μM), was infiltrated to the same location 12 h prior to the collection time points.

### 4.6. Transient Expression of Fluorescent Chimeras of HPE1 and BcRAD23 in Cultured Tni Insect Cells

Constructs encoding fluorescent chimeras of HPE1B mature protein and BcRAD23 tagged at their C-terminal ends with mCherry or EGFP (enhanced green fluorescent protein) were generated via overlap extension PCR [47], and then cloned into the pIB/v5-His insect cell expression vector (Thermo Fisher Scientific, Waltham, MA, USA). Overlap extension PCR was performed using KOD-Hot Start DNA polymerase (EMD Millipore, San Diego, CA, USA) as described [48] with sequence-validated plasmids and primers listed in Table S1. Initial thermocycler conditions consisted of 95 °C for 2 min followed by 25 cycles at 95 °C for 20 s, 58 °C for 20 s, 70 °C for 1:30 min, and a final extension at 70 °C for 5 min. Final thermocycler conditions consisted of 95 °C for 2 min followed by 27 cycles at 95 °C for 20 s, 56 °C for 20 s, 70 °C for 1:30 min, and a final extension at 70 °C for 5 min. Clones were sequence verified (Retrogen Inc., San Diego, CA, USA).

Cultured *Trichoplusia ni* (Tni) cells (Allele Biotech Inc., San Diego, CA, USA), an embryonic insect cell line [49], were transfected on 35-mm #1.5 glass-bottom dishes (Matsunami Glass USA Inc., Bellingham, WA, USA) as described [48] using Cellfectin II (Thermo Fisher Scientific Waltham, MA, USA). Localization of the transiently expressed chimeras was examined 48 hr post-transfection using a 60× phase contrast water immersion objective/NA 1.2 on a FluoView FV10i laser scanning confocal microscope (Olympus Scientific Solutions, Waltham, MA, USA). Images were processed using Photoshop 21.2.9 (Adobe Inc., Mountain View, CA, USA).

### 4.7. Protein Extraction and Immunoblotting

Plant samples were homogenized in protein extraction buffer [10 μM Tris-HCL, 0.5 μM EDTA, 0.4 μM DTT, 1X Protease inhibitor cocktail (Thermo Fisher scientific, Waltham, MA, USA)]. Total protein extracts were quantified by Bradford assay. Around 10 μg of proteins mixed with 4X SDS sample buffer were loaded onto each lane of a 4–12% Bis-Tris NuPage gel (Invitrogen, Carlsbad, CA, USA). Proteins were transferred to Immobilon-P PVDF membrane (Millipore-Sigma, Burlington, MA, USA) using standard blotting protocol. Ponceau S staining was used to visualize protein loading. The membrane was then blocked with 5% dry milk in TBST buffer followed by incubation with the desired primary antibody [anti-HA (Invitrogen, Carlsbad, CA, USA), anti-Ubiquitin (Cytoskeleton Inc., Denvor, CO, USA), or anti-FLAG (Millipore-Sigma, Burlington, MA, USA)] at 4 °C overnight. After washing off the primary antibody, the blot was incubated with HRP-conjugated goat anti-mouse IgG (H+L) secondary antibody (Invitrogen, Carlsbad, CA, USA) at room temperature for 1 h. The bound antibody was detected using the SuperSignal West Pico substrate (Invitrogen, Carlsbad, CA, USA) and imaged on an iBright 1500 imaging system (Thermo Fisher Scientific, Waltham, MA, USA). Signal intensity was analyzed and quantified using the iBright Analysis Software (Thermo Fisher Scientific, Waltham, MA, USA). Means of signal intensity were compared with Student’s *t*-test.

### 4.8. Generation of Transgenic Plants

The sequences encoding the full-length or mature protein of HPE1B were cloned with *Bam*HI and *Kpn*I sites into the binary vector pERGFP-1380N, generating pHPE1-FL-OE, and pHPE1-MP-OE constructs under the control of the Cauliflower mosaic virus 35S promoter. The constructs were sequenced to confirm the correct insertion of the HPE1B and in-frame HA fusion tags. Transgenic tomatoes were generated at the Multi-Crop Transformation Facility at Texas A&M University using previously described methods [50,51]. Seedlings generated from calli grew on selection media (Kanamycin) were separated into individual tubes with rooting media, and later screened by PCR using HPE1 specific primers.

Individual T_0_ plants were tested for HPE1 gene expression by RT-PCR and for protein expression by dot blot using an anti-HA antibody (Invitrogen, Carlsbad, CA, USA). T_0_ cutting plants were generated from the axillary shoots, and allowed to root for one week before transplanting. Two-week-old cutting plants expressing the HPE1 gene were used for Lso infection experiments. For each plant, four adult psyllids from the LsoB population were allowed to feed for seven days before being removed from the plant. Leaf samples from the second-tier leaves were collected weekly from 2 to 5 weeks postinfection to detect Lso cells by PCR as previously described. Wild-type plants were treated similarly.

## Data Availability

The data that support the findings of this study are available upon resonable request made to the corresponding author.

## Supplementary Materials

The following are available online at https://www.mdpi.com/article/10.3390/ijms22169003/s1, Table S1: Primers used in this study.
