# Supplementary material for: HPE1, an Effector from Zebra Chip Pathogen Interacts with Tomato Proteins and Perturbs Ubiquitinated Protein Accumulation
Source: Int J Mol Sci. 2021 Aug 20;22(16):9003. doi: 10.3390/ijms22169003 (PMC8396652; doi:10.3390/ijms22169003)
Supplement: Supplementary file 1 [file ijms-22-09003-s001.zip › ijms-1317431-supplementary.pdf]

Table S1. Primers used in this study.

Primer	Sequence (5'-3')	Purpose
SlRADa-AD-F	CACCCATATGAAGCTCACTGTAAAGACTCTC	SlRAD23a Y2H prey cloning
SlRADa-AD-R	CGTATCGATATCTTCATAATCTCCTGCATGC	SlRAD23a Y2H prey cloning
SlRADc-AD-F	CACCCATATGAAGATTTTTGTGAAAACTCTC	SlRAD23c Y2H prey cloning
SlRADc-AD-R	CGTATCGATCTCTTCAAACTCGTGAATGTGA	SlRAD23c Y2H prey cloning
SlRADd-AD-F	CACCCATATGAAGATTTTTGTGAAGACTTTG	SlRAD23d Y2H prey cloning
SlRADd-AD-R	CGTATCGATTTCATCAAACTCATGCATGTG	SlRAD23d Y2H prey cloning
SlRADe-AD-F	GCGGAATTCATGAAGATTTTCGTGAAGAC	SlRAD23e Y2H prey cloning
SlRADe-AD-R	GCGGGATCCTCACTCGTCAAACTCATGCAAG	SlRAD23e Y2H prey cloning
BcRAD-AD-F	GCGGAATTCATGAAGCTAACGATTAAAAATC	BcRAD23 Y2H prey cloning
BcRAD-AD-R1	ATCGCTCGAGGTCGTCCATGTTTTGCGAGAG	BcRAD23 Y2H prey cloning
BcRAD-AD-R2	GCGGGATCCAGGCTTTGGTTTGGACAACAT	BcRAD23-UBL prey cloning
HPE1A-BD-F	CACCGAATTCATGCACCATCCCATTAAGACAC	HPE1-A Y2H Bait cloning
HPE1A-BD-R	GCGGGATCCTTAGTGACGCGCAGAATCA	HPE1-A Y2H Bait cloning
HPE1B-BD-F	CACCGAATTCATGCACCATCCTATTAGGACAC	HPE1-B Y2H Bait cloning
HPE1B-BD-R	GCGGGATCCTTACTGATGCGCAGAATCC	HPE1-B Y2H Bait cloning
SlRADe-GW-R	CTCGTCAAACTCATGCAAGTGA	SlRAD23e pENTR cloning
HPE1B-GW-R	CTGATGCGCAGAATCCTTAA	HPE1-B pENTR cloning
Lso-F	CGAGCGCTTATTTTTAATAGGAGC	Lso infection diagnostic
Lso-R	GCCTCGCGATTCGCAACCCAT	Lso infection diagnostic
EF1alpha-F	GAAGGTCGGTTACAACCCTGAC	EF1α housekeeping gene
EF1alpha-R	GAGCATATCCGTTTCCAATCTG	EF1α housekeeping gene
HPE1B-qF	TGTCAAATGTCTAAGTTGCGAATTG	HPE1 T_0 gene expression
HPE1B-qR	ATCGGTCATGACTATGGCATTT	HPE1 T_0 gene expression
BcRAD23 start F	ATGAAGCTAACGATTAAAAATCTTC	BcRAD23 OE-PCR
BcRAD23-mCherry F	CAAAACATGGACGACATGGTGAGCAAGGGC	BcRAD23 OE-PCR
BcRAD23-mCherry R	GCCCTTGCTCACCATGTCGTCCATGTTTTG	BcRAD23 OE-PCR
mCherry R	CTACTTGTACAGCTCGTC	BcRAD23 OE-PCR
Lso4420B start F	ATGCAGTCAGTTTATGAAATACAC	HPE1B OE-PCR
Lso4420B-EGFP F	GATTCTGCGCATCAGATGGTGAGCAAGGGC	HPE1B OE-PCR
Lso4420B-EGFP R	GCCCTTGCTCACCATCTGATGCGCAGAATC	HPE1B OE-PCR
EGFP R	GACTTGTACAGCTCGTCC	HPE1B OE-PCR
